# Renal cell carcinoma with unusual metachronous metastasis up to 22 years after nephrectomy: two case reports

**DOI:** 10.1186/s13256-021-03098-5

**Published:** 2021-10-05

**Authors:** F. Bruckschen, C. D. Gerharz, A. Sagir

**Affiliations:** 1 Department of Gastroenterology, Academic Teaching Hospital Bethesda Duisburg, Heerstr. 219, 47053 Duisburg, Germany; 2 Department of Pathology, Academic teaching Hospital Bethesda Duisburg, Heerstr. 219, 47053 Duisburg, Germany

**Keywords:** Renal cell carcinoma, Metastasis, Pancreas, Omentum, Liver

## Abstract

**Introduction:**

Renal cell carcinoma is the third most common malignant tumor in the urogenital tract. An estimated 25% of renal cell carcinomas are in stage IV when diagnosed. The 5-year-survival with stage IV is about 20%. Late metastases are found after an extended disease-free interval up to 20 years after primary nephrectomy.

**Case presentation:**

Here, we present two cases with late-onset metastasis of renal cell carcinoma with different clinical presentations. The first patient, an 88-year-old Caucasian man, presented with bleeding of the upper gastrointestinal tract. Biopsies taken from the duodenal bulb showed a tumor compatible with a solitary metastasis from renal cell carcinoma 22 years ago. The second patient, a 79-year-old Caucasian man, consulted our gastroenterological department with results of an outpatient computed tomography scan with multiple suspected tumor areas in the liver, omentum, thyroid, and mediastinum. A computed tomography-guided liver biopsy was performed that showed a clear-cell tumor consistent with a metastasis of the renal cell carcinoma 17 years ago.

**Conclusion:**

Both cases show that patients with a history of renal cell carcinoma should be followed up for a longer time than patients with other malignant tumors.

## Background

Renal cell carcinoma (RCC) is the third most common malignant tumor in the urogenital tract. Two of three patients are men. RCC commonly metastasizes to the lung parenchyma (45.2%), skeleton (29.5%), lymph nodes (20.8%), liver (20.3%), adrenals (8.9%), and brain (8.1%) [1]. The most important risk factors are smoking, obesity, and hypertension. It was shown that there is a clear dose–effect relationship between the risk factors and development of RCC. Heavy smokers who are overweight and have high blood pressure have the highest risk for developing RCC [2–4]. In addition, hereditary diseases like von-Hippel–Lindau syndrome also play a role [5].

An estimated 25% of RCCs are in stage IV when they are diagnosed. Also every fourth patient presented with M1 (that is, metastasis) after primary nephrectomy. The 5-year survival in stage IV is only 12%.

Literature shows that metastatic RCC is characterized by an extended disease-free interval up to 20 years after primary nephrectomy [6]. Typically, metastases are found in the lung, bone, and liver. In these metastatic stages, a target therapy is first-line therapy [7]. Metastases into the pancreas are rare and found mostly in asymptomatic patients [8]. In the case of a solitary pancreatic metastasis from a RCC, resection is recommended to prolong the overall survival [9]. Other atypical RCC metastases into the omentum, thyroid, and mediastinum are very rare. Here, we report two cases of RCC metastasis at least 17 years after nephrectomy.

## Case presentation

### Case 1

An 88-year-old Caucasian man presented to our emergency department with gastrointestinal bleeding [hemoglobin (Hb) 8.3 g/dl]. No other symptoms were documented. A history of Alzheimer’s disease, renal cell carcinoma 21 years ago with nephrectomy and thyroid metastasis 12 years ago, paroxysmal atrial fibrillation, and diabetes mellitus type II were known. The patient was conscious, disoriented, and in debilitated general condition. He appeared to be in a good nutritional state (height 164 cm, weight 62 kg, body mass index 23.1 kg/m^2^). His lungs were clear to auscultation and percussion bilaterally. Examination of his heart showed an irregular heartbeat. The abdomen was soft, moderately distended, and nontender to palpation. An esophagogastroscopy was performed immediately with detection of an ulcer in the duodenal bulb (Forrest III). Medical history included a vitamin K antagonist, so that the Quick’s value was 21% and biospy could not be taken caused by the low Quicks´s value. The patient had a fall the following night and suffered a dislocated pertrochanteric femur fracture. He was transferred to the department for orthopedic surgery where the fracture was treated. Following complex geriatric management, including treatments by physiotherapists accompanied by specialized nurses, he could leave the hospital.

**Fig. 1:**
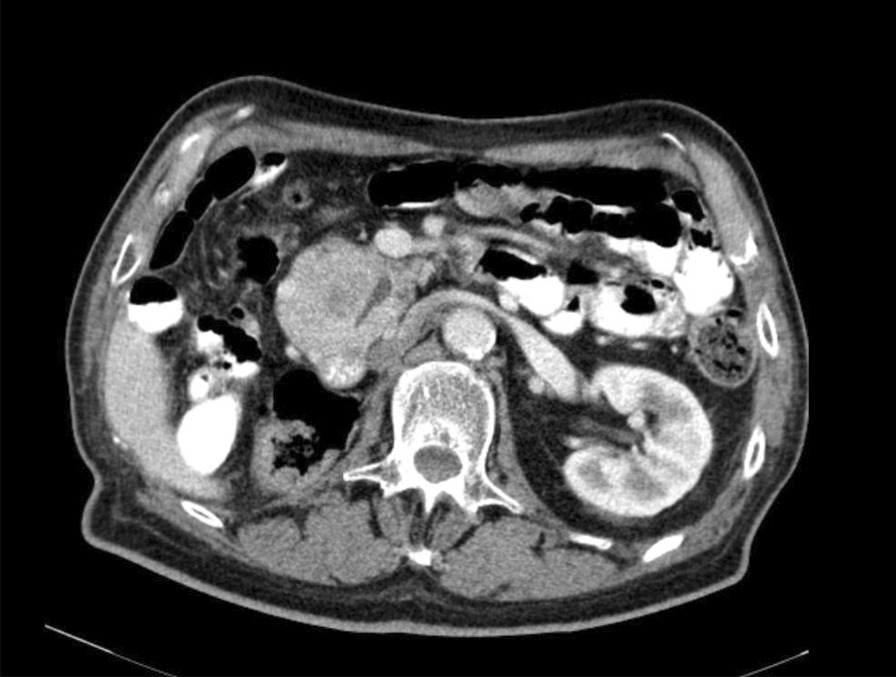
Axial contrast-enhanced computed tomography demonstrating tumor of the pancreatic head with stenosis of the main pancreatic duct

One year before the consultation, he had undergone a computed tomography (CT) scan that showed a suspected tumor area in the pancreatic head with questionable infiltration of the duodenum and the inferior mesenteric vein (Fig. 1). In addition, there was a blocking of the pancreatic duct. CT diagnosis of suspicious malignancy of the pancreas was suspected. A biopsy to confirm the suspected diagnosis was not performed. At this time carcinoembryonic antigen (CEA) and cancer antigen 19-9 (CA 19-9) were within the normal ranges.

Ten months later, the patient presented to our emergency department with Hb-relevant gastrointestinal bleeding (Hb 9.0 g/dl) again. Vitamin K antagonist was changed to apixaban. The patient was hemodynamically stable, so an esophagogastroscopy was performed 2 days later. Apixaban was paused when he presented to the emergency department. Treatment given to the patient included levothyroxine, pantoprazole, metoprolol, torasemide, levodopa, metamizole, and macrogol. A duodenal bulb infiltration of the suspected pancreatic tumor was supposed. Biopsies were taken from the duodenal bulb.

The medical history of the patient revealed right nephrectomy 22 years ago due to to renal cell carcinoma. The patient underwent total thyroidectomy because of a solitary metastasis from the neoplasia 12 years ago.

### Laboratory findings at first presentation

A routine laboratory analysis of the first patient showed the following abnormal values: hemoglobin (Hb) 8.3 (14.0–18.0) g/dl, hematocrit 26.4% (36–49%), mean corpuscular volume (MCV) 99.2 (79–96) fl, folic acid > 40.00 (5–19) ng/ml, creatinine 1.87 (0.67–1.17) mg/dl, urea 86 (18–55) mg/dl, uric acid 7.7 (3.2–7.4) mg/dl, random serum glucose 119 (74–106) mg/dl, total protein 5.61 (6.60–8.30) g/dl, transferrin 154 (200–360) mg/dl, reticulocytes 2.93% (0.80–2.50%), immature reticulocytes 12.9% (0.0–2.0%), thyroid stimulating hormone (TSH) 82.43 (0.53–3.59) µU/ml, free triiodothyronine (fT3) 1.13 (2.28–5.01) pg/ml, free tetraiodothyronine (fT4) 0.46 (0.93–1.60) ng/dl, gamma glutamyl transferase (GGT) 82 (< 54) U/l, and alkaline phosphatase (AP) 249 (40–130) U/l.

### Histology

**Fig. 2:**
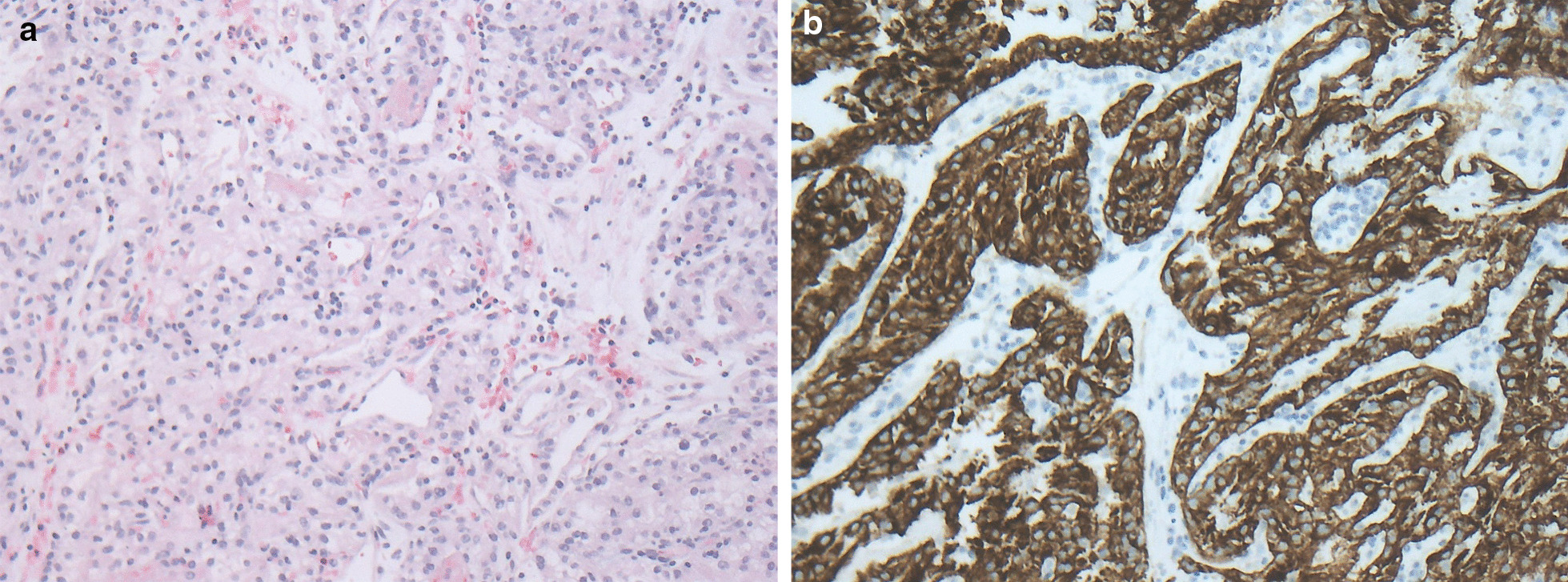
Duodenal ulcer of patient 1 with slightly eosinophilic solid tumor cell complexes (**a**; hematoxylin and eosin), exhibiting an intensive brown immunostaining (**b**) for cytokeratin

Histological examination showed a solid, partly pseudoglandular clear-cell carcinoma in the biopsy, compatible with a solitary metastasis from the renal cell carcinoma 22 years ago (Fig. 2).

### Case 2

**Fig. 3:**
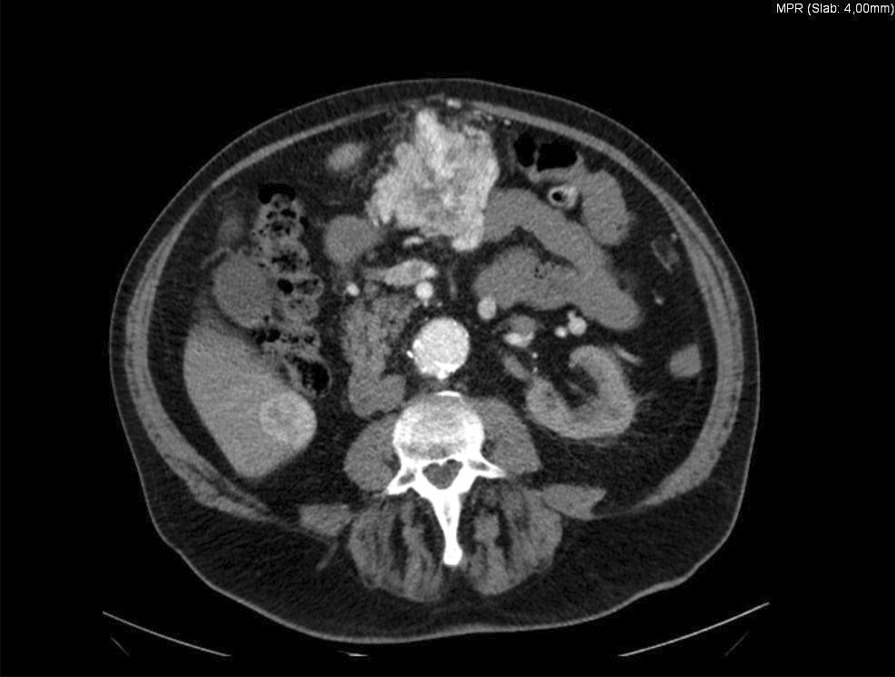
Arterial phase of CT scan showing hypervascular tumor in a right caudal liver segment as well as intraperitoneal in the upper abdomen

A 79-year-old Caucasian man presented to our gastroenterological department with results of an outpatient CT scan. He had undergone this examination because of an abnormal ultrasound showing enlarged lymph nodes located along the aorta. The radiological examination showed suspected tumor areas in the liver, omentum, thyroid, and mediastinum (Fig 3). The medical history of this patient revealed a right-sided nephrectomy 17 years ago due to a renal cell carcinoma. He was also known to have arterial hypertension, diabetes mellitus type II, and infrarenal aortic aneurysm.

### Physical examination

On general physical examination, the patient was conscious and oriented in fair general condition. He appeared to be in a good nutritional state (height 178 cm, weight 86 kg, body mass index 27.1 kg/m^2^). Lungs were clear to auscultation and percussion bilaterally. Heart examination was also normal. He had a soft, nontender abdomen without any palpable masses. Icterus and lymphadenopathy were absent. Vital signs were normal.

### Laboratory findings

The following measurements were made: hemoglobin (Hb) 13.3 (14.0–18.0) g/dl, hematocrit 40.3% (36–49%), mean corpuscular volume (MCV) 91.0 (79–96) fl, creatinine 1.24 (0.67–1.17) mg/dl, urea 40 (18–55) mg/dl, uric acid 8.1 (3.2–7.4) mg/dl, random serum glucose 114 (74–106) mg/dl, total protein 7.17 (6.60–8.30) g/dl, thyroid stimulating hormone (TSH) 0.12.43 (0.53–3.59) µU/ml, free triiodothyronine (fT3) 3.94 (2.28–5.01) pg/ml, and free tetraiodothyronine (fT4) 1.75 (0.93–1.60) ng/dl. Transaminases and cholestasis parameter were normal.

## Imaging modalities and histology

Ultrasound scan showed an aortic aneurysm, a diffuse slightly arterial hypervascular lesion in the middle abdomen and in segments 5/6 of the liver, steatosis hepatis, and a nephrectomy of the right kidney. The patient underwent esophagogastroscopy and colonoscopy to search for a primary tumor. A chronic peptic esophagitis and gastritis could be detected by esophagogastroscopy. Colonoscopy was without any pathological finding. Endoscopic ultrasound showed an intraperitoneal mass of 8 cm diameter in the upper abdomen without any association to an organ. The mass exhibited low echogenicity and showed a slightly arterial hypervascular enhancement.

**Fig. 4:**
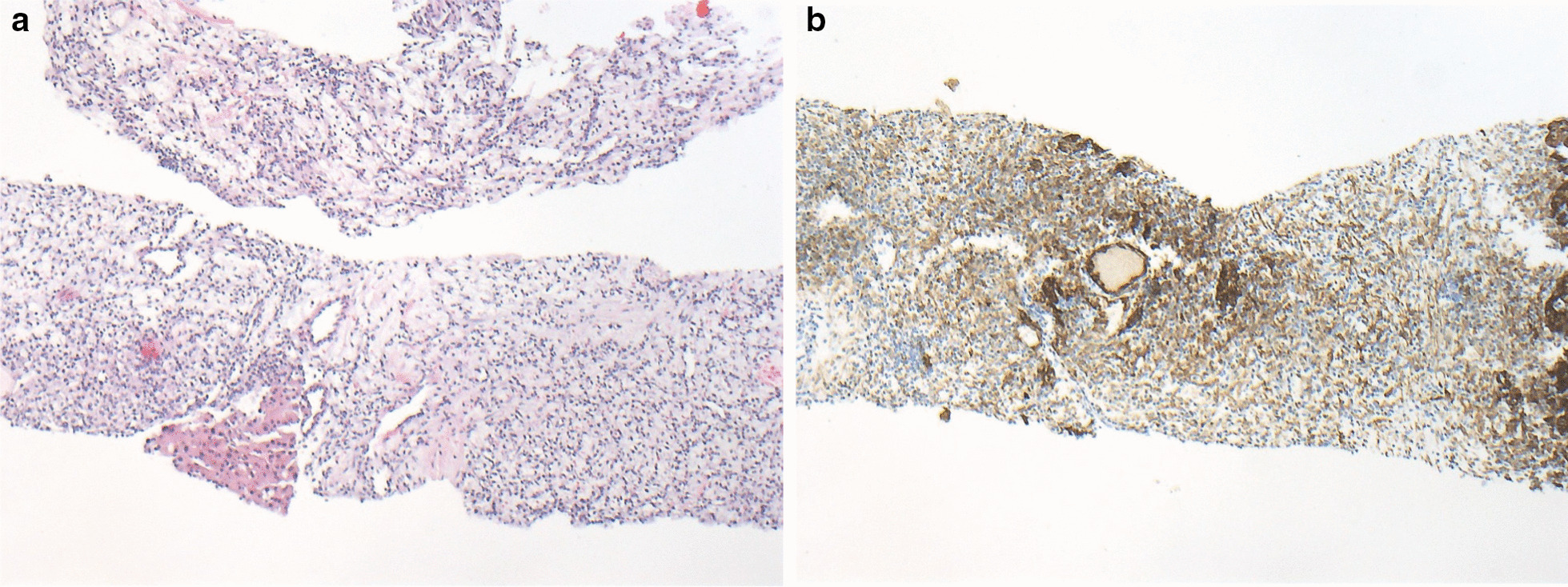
Liver biopsy of patient 2 with extensive solid tumor cell complexes, exhibiting clear-cell cytoplasm (**a**; hematoxylin and eosin), and an intensive brown immunostaining (**b**) for cytokeratin

In the next step, the patient underwent CT-guided biopsy of the liver. The histology of the liver biopsy from the second patient also showed a clear-cell carcinoma with expression of vimentin and cytokeratin (Fig. 4). These findings are also consistent with multiple late metastases from the primary, surgically treated renal tumor 17 years ago.

### Further process

In consultation with the wife of the first 88-year-old patient, we decided to start palliative care in favor of the recommended surgical procedure. Two months after this diagnosis, our patient died in a retirement home.

The second patient was presented to our tumor board, with the recommendation to start chemotherapy with the tyrosine kinase inhibitor pazopanib. The patient showed abdominal progression 1 year after initiation of therapy with pazopanib, so the therapy was changed to nivolumab. A CT scan 6 months after the switch from pazopanib to nivolumab again showed abdominal progression. Therapy with cabozantinib was started.

## Discussion and conclusions

RCC is a urological carcinoma that accounts for 3% of all adult malignancies, and is the most common renal malignancy that originates in the lining of the proximal convoluted tubules [10]. Nephrectomy is the curative treatment of RCC. The malignancy is known to metastasize several years after the primary tumor has been treated [11]. RCC metastasis includes the lung and bone, while lymph nodes, brain, liver, and contralateral kidney are less common sites. Metastasis to the pancreas and gastrointestinal tract is rare.

The first case presented in our department with an upper gastrointestinal bleeding. The patient had undergone CT scan some months earlier with suspicion of a pancreatic tumor, which could be confirmed as a pancreatic metastasis of his RCC 22 years after initial diagnosis on biopsies taken from the duodenal bulb ulcer.

Secondary neoplasms affecting the pancreas are uncommon. Only 2–5% of the malignancies of the pancreas are not primary pancreatic tumors [12]. Pancreatic metastasis derives from renal cell carcinomas, melanomas, colorectal carcinomas, lung carcinomas, breast carcinomas, and sarcomas [13]. Based on the advances made in pancreatic surgery, attention has been paid especially to metastatic RCC affecting the pancreas [8]. Unlike other malignancies affecting the pancreas secondarily, which are often associated with widespread systemic disease, RCC may spread to the pancreas as the only secondary site. This makes the surgical resection of isolated RCC of the pancreas an attractive form of therapy. While histological proof of a primary pancreatic carcinoma that undergoes surgical therapy is not recommended, an accurate histological diagnosis of RCC spreading secondarily to the pancreas is necessary for the management.

One year before the first consultation, the patient underwent a CT scan with detection of a tumor in the pancreas. Retrospectively, this tumor had been misinterpreted as a primary pancreatic tumor. Considering the prior history of RCC in this patient, percutaneous or endoscopic ultrasound-guided biopsy of the pancreatic tumor had been legitimate. Due to the extremely low prevalence of pancreatic metastasis of RCC, the colleagues had not considered this diagnosis more than 20 years after the nephrectomy. Pancreatic metastases do not usually represent a challenging diagnosis as they normally develop early in the presentation of a known primary malignancy. An exception is RCC, which is notable for metastasizing long after the diagnosis and treatment of the primary tumor, with a mean interval between primary RCC and pancreatic metastasis of 8–10 years [14]. In our case, the RCC was treated more than 20 years ago. We think that this case is one of the latest manifestations of a pancreatic metastasis ever reported. Alayyaf *et al*. report a case with very late pancreatic metastasis of RCC 8 years after radical nephrectomy [15]. Their patient was asymptomatic and underwent a spleen-preserving distal pancreatectomy.

The second patient consulted our department with the results of a CT scan. This CT scan was performed after an ultrasound examination due to his known aortic aneurysm. The radiological examination showed suspected tumor areas in the liver, omentum, thyroid, and mediastinum. The patient was totally asymptomatic. While metastasis of RCC in the liver and mediastinum is frequently observed, omental metastasis is very rare [16]. Metastasis to the thyroid is relatively rare, despite its rich blood supply. RCC, however, is one of the more common types of neoplasms to metastasize to the thyroid. However, little is known about the mechanism of metastasis or the potentially advantageous environment of the thyroid for RCC cells [17]. Diagnosis of metachronous metastasis from RCC after 17 years ago was done by CT-guided biopsy. Disseminated metastases in the abdomen, thorax, and neck long after diagnosis and treatment are very unusual. Treatment with a tyrosine kinase inhibitor is the recommended therapy in this situation.

In summary, we report two cases of metachronous metastasis more than 17 years after nephrectomy due to RCC. Although metastasis from RCC is not unusual, both cases are very unusual. The first case presented with a single pancreatic metastasis 22 years after nephrectomy. The metastasis had been misinterpreted as a primary pancreatic tumor, and metastasis was diagnosed only after upper-gastrointestinal bleeding. The second case showed disseminated metastasis in the abdomen, thorax, and neck 17 years after nephrectomy. Based on the late onset of metastasis, patients with RCC in their medical history need follow-up for a longer time than patients with other malignancies.

## Data Availability

All data and images are in the manuscript.
